# Relationship between interpersonal sensitivity and leukocyte telomere length

**DOI:** 10.1186/s12881-017-0473-9

**Published:** 2017-10-10

**Authors:** Akihito Suzuki, Yoshihiko Matsumoto, Masanori Enokido, Toshinori Shirata, Kaoru Goto, Koichi Otani

**Affiliations:** 1 Department of Psychiatry, Yamagata University School of Medicine, 2-2-2 Iidanishi, Yamagata, 990-9585 Japan; 2 Department of Anatomy and Cell Biology, Yamagata University School of Medicine, 2-2-2 Iidanishi, Yamagata, 990-9585 Japan

**Keywords:** Interpersonal sensitivity, IPSM, Telomere length

## Abstract

**Background:**

Telomeres are repetitive DNA sequences located at the ends of chromosomes, and telomere length represents a biological marker for cellular aging. Interpersonal sensitivity, excessive sensitivity to the behavior and feelings of others, is one of the vulnerable factors to depression. In the present study, we examined the effect of interpersonal sensitivity on telomere length in healthy subjects.

**Methods:**

The subjects were 159 unrelated healthy Japanese volunteers. Mean age ± SD (range) of the subjects was 42.3 ± 7.8 (30–61) years. Interpersonal sensitivity was assessed by the Japanese version of the Interpersonal Sensitivity Measure (IPSM). Leukocyte telomere length was determined by a quantitative real-time PCR method.

**Results:**

Higher scores of the total IPSM were significantly (*β* = −0.163, *p* = 0.038) related to shorter telomere length. In the sub-scale analysis, higher scores of timidity were significantly (*β* = −0.220, *p* = 0.044) associated with shorter telomere length.

**Conclusions:**

The present study suggests that subjects with higher interpersonal sensitivity have shorter leukocyte telomere length, implying that interpersonal sensitivity has an impact on cellular aging.

## Background

The personality trait of interpersonal sensitivity is defined as undue and excessive awareness of, and sensitivity to, the behavior and feelings of others [1]. Individuals with this personality are preoccupied with their interpersonal relationships, vigilant to the behavior and moods of others, and overly sensitive to perceived or actual criticism or rejection and their behavior is modified with other’s expectations to minimize the risk of criticism or rejection. It has been widely accepted that interpersonal sensitivity is a risk factor for depression, especially postnatal depression [2, 3], non-melancholic depression [4], and atypical depression [5]. This personality has also been related to bulimic symptomatology in undergraduate students [6], nicotine dependence in a community sample [7], and alcohol dependence in depressive patients [8]. Recent studies show that the function of hypothalamic-pituitary-adrenal (HPA) axis is involved in the characterization of this personality [9–12].

Telomeres are repetitive DNA sequences located at the ends of chromosomes and play a crucial role in preventing chromosome fusion and in maintaining genome stability [13, 14]. Telomere length is maintained by telomerase, a cellular enzyme, in germ cells and stem cells, while most somatic cells have very low telomerase activity, thus leading to telomere length shortening with cellular division [13, 14]. When telomere length reaches a critical point, cellular senescence is triggered, cell division ceases, and the cell dies [13, 14]. Thus, telomere length represents a biological marker for cellular aging. Previous studies have shown that telomere length is related to coronary heart disease [15, 16], cancer [17, 18], progression of diabetic nephropathy in patients with type 1 diabetes [19], dementia in post-stroke patients [20], and mortality [21, 22]. Shorter telomere length is also associated with mood disorders, schizophrenia, mild cognitive impairment, and Alzheimer disease [23].

It has been suggested that telomere length is influenced by certain personality traits such as pessimism [24, 25], neuroticism [26], and hostility [27]. We hypothesized that interpersonal sensitivity might be one of the factors influencing telomere length; however, there is no study examining the effects of interpersonal sensitivity on telomere length. Therefore, in the present study, we examined the relationship between interpersonal sensitivity and leukocyte telomere length in healthy subjects.

## Methods

Originally, 192 physically healthy Japanese were recruited from hospital staffs. Exclusion criteria were the presence of physical diseases, which was assessed by using the self-reported check sheets for physical diseases and treatments, and the presence of a current or past history of psychiatric disorders according to the Diagnostic and Statistical Manual of Mental Disorders-IV [5]. Psychiatric screening was conducted by interviews by well trained psychiatrists and a questionnaire on psychiatric treatment and diagnosis. Nine cases had psychiatric disorders and 18 had missing data. Data of 6 subjects were excluded due to failure of DNA extraction or PCR amplification. The remaining 159 subject samples were used for analysis. Mean age ± SD (range) of the subjects was 42.3 ± 7.8 (30–61) years. The subject’s characteristics are shown in Table 1. The study protocol was approved by the ethics committee of the Yamagata University School of Medicine. After complete description of the study to the subjects, written informed consent was obtained from all subjects.

**Table 1:** Characteristics of subjects, relative telomere length, and IPSM scores

Number of subjects (n)	159
Male/Female (n)	83/76
Age (years, mean ± SD (range))	42.3 ± 7.8 (30–61)
Relative telomere length (z-score, range)	−2.05 - 3.54
IPSM (score, mean ± *SD*)	
Total	64.6 ± 10.7
Interpersonal awareness	18.0 ± 3.5
Separation anxiety	17.5 ± 3.8
Timidity	19.6 ± 2.9
Fragile inner-self	9.5 ± 2.3

*IPSM* Interpersonal Sensitivity Measure, *SD* Standard deviation

Interpersonal sensitivity was measured by the Japanese version of the Interpersonal Sensitivity Measure (IPSM), which has been verified to have high test-retest reliability, item-total correlation, internal consistency, and discriminant validity [28]. The IPSM developed by Boyce and Parker [1] and later modified by Boyce et al. [29] is a self-report scale with 28 items to assess this personality trait. The IPSM has 4 sub-scales, i.e., interpersonal awareness which refers to vigilance to the behavior and feelings of others, separation anxiety which deals with anxiety about separation from significant others, timidity which assesses lack of assertiveness for fear of upsetting others, and fragile inner-self which identifies difficulty with self-disclosure for fear of rejection. In the present sample, the values of Cronbach’s α for total IPSM, interpersonal awareness, separation anxiety, timidity, and fragile inner-self were 0.911, 0.818, 0.817, 0.674, and 0.706, respectively, suggesting that the magnitudes of these values were considered to be satisfactory.

Ten ml of blood was obtained from subjects’ median vein in a EDTA-containing tube, and genomic DNA was extracted from peripheral leukocytes using a QIAamp DNA Blood Kit (Qiagen, Tokyo, Japan), and was stored at −80 °C before PCR amplification. Leukocyte relative telomere length, assessed by a ratio of telomere/single copy gene (36B4) with the mean data from the triplicate runs, was determined by a quantitative real-time PCR method of Cawthon [30] with several modifications [31]. The intra- and inter-assay coefficients of variation for the telomere reaction were 1.67% and 1.71%, respectively, and those for the 36B4 reaction were 3.19% and 5.06%, respectively. Relative telomere length was expressed as a standardized z-score in this study, since a standard curve to perform quantification of each DNA sample was created from DNA dilution of a subject, i.e., obtained telomere length was expressed as relative length to the subject. The telomere length was determined by researchers who were blind to the results of the IPSM.

In our previous studies, relative telomere length was influenced by gender and age [31, 32]. Thus, the effects of the total and 4 sub-scale scores of the IPSM on telomere length were tested by the multiple regression analysis with telomere length as a dependent variable and with the IPSM scores, age, and gender as independent variables. No elimination technique was adopted, and all the independent variables were used. A dummy variable was used for gender (female = 0, male = 1). Sex differences in the IPSM scores and telomere length were tested by the Student t-test. Normality of data distribution was checked using the Kolmogorov-Smirnov test. The Smirnov-Grubbs’ test was used to evaluate outliers of telomere length. All statistical analyses were performed by SPSS 14.0 J for Windows (SPSS Japan Inc., Tokyo, Japan), and a *p* value of less than 0.05 (two-tailed) was regarded as significant.

## Results

The effects of the total and 4 sub-scale scores of the IPSM on telomere length are shown in Table 2 and Table 3, respectively. The data for telomere length was normally distributed (*p* = 0.079), and did not involve outliers. In the present sample, there were no sex differences in the IPSM scores (male vs. female, mean ± SD: Total IPSM, 64.0 ± 9.1 vs. 65.2 ± 12.2, *p =* 0.494; interpersonal awareness, 17.7 ± 3.3 vs. 18.3 ± 3.8, *p =* 0.304; separation anxiety, 17.5 ± 3.6 vs. 17.6 ± 4.1, *p =* 0.877; timidity, 19.6 ± 2.6 vs. 19.6 ± 3.2, *p =* 0.997; fragile inner-self, 9.3 ± 1.7 vs. 9.8 ± 2.7, *p =* 0.158) nor telomere length (−0.11 ± 0.93 vs. 0.12 ± 1.06, *p =* 0.147).

**Table 2:** Effects of the total IPSM scores, age, and sex on relative telomere length

	Relative telomere length	
	*β*	*p*
Total IPSM	−0.163	0.038
Age	−0.169	0.031
Sex	−0.126	0.106
Multiple correlation coefficient	*R* = 0.264, *p* = 0.011	

*IPSM* Interpersonal Sensitivity Measure

The multiple regression analysis was performed with telomere length as a dependent variable and with the IPSM scores, age, and gender as independent variables

**Table 3:** Effects of the 4 sub-scale scores of the IPSM, age, and sex on relative telomere length

	Relative telomere length	
	*β*	*p*
Interpersonal awareness	−0.053	0.656
Separation anxiety	0.055	0.656
Timidity	−0.220	0.044
Fragile inner-self	0.018	0.874
Age	−0.159	0.042
Sex	−0.119	0.133
Multiple correlation coefficient	*R* = 0.301, *p* = 0.024	

*IPSM* Interpersonal Sensitivity Measure

The multiple regression analysis was performed with telomere length as a dependent variable and with the IPSM scores, age, and gender as independent variables

**Fig. 1:**
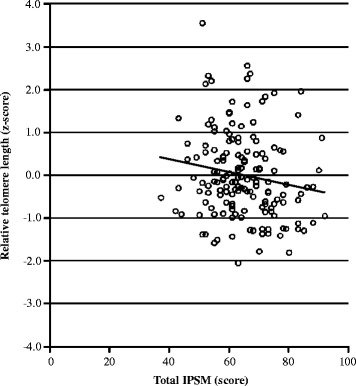
Relationship between interpersonal sensitivity and telomere length. IPSM; Interpersonal Sensitivity Measure

Higher scores of the total IPSM (*β* = −0.163, *p* = 0.038) were significantly related to shorter telomere length (Table 2 and Fig. 1). Age was negatively correlated with telomere length (*β* = −0.169, *p* = 0.031) (Table 2).

In the sub-scale analysis, higher scores of timidity (*β* = −0.220, *p* = 0.044) were associated with shorter telomere length, while the scores of interpersonal awareness, separation anxiety, and fragile inner-self were not related to telomere length (Table 3). Age was negatively correlated with telomere length (*β* = −0.159, *p* = 0.042) (Table 3).

## Discussion

This study was the first attempt to examine the relationship between interpersonal sensitivity and telomere length. In the present study, there was a significant negative correlation between the total IPSM scores and leukocyte telomere length, i.e., the subjects who have excessive sensitivity to other’s behaviors and feelings were more likely to have shorter telomere length. The present result is in line with the previous study reporting that high neuroticism, which is related to interpersonal sensitivity [33], was associated with shorter telomere length [26].

Several mechanism(s) might explain the association between interpersonal sensitivity and telomere length observed in the present study. Firstly, it has been reported that high interpersonal sensitivity is associated with health-harming behaviors such as bulimic behavior [6], nicotine dependence [7], and alcohol dependence [8]. These behaviors have been associated with telomere length shortening [14], thus high interpersonal sensitivity may influence telomere shortening through these health-harming behaviors. Secondly, it has been reported that subjects with high levels of interpersonal sensitivity-related traits such as neuroticism and rejection sensitivity have an increased HPA activity [11, 12, 34], although the finding is inconsistent [9, 10]. An in vitro study showed that exposure of human T lymphocytes to cortisol caused a reduction in telomerase activity [35], and in vivo, elevated urinary nocturnal cortisol levels were associated with shorter telomere length in healthy women [36]. Therefore, it is possible that the association between high interpersonal sensitivity and short telomere length is mediated by elevated cortisol levels.

There are several limitations in the present study. Firstly, the present study was a cross-sectional design, i.e., interpersonal sensitivity and telomere length were assessed simultaneously. Thus, the causal relationship between interpersonal sensitivity and telomere length remains unclear. Secondly, the sample size of the present study was relatively small. In addition, all subjects were Japanese hospital staffs, suggesting that replication study is needed with large number of subjects from general populations or other ethnic groups. Thirdly, the present study did not assess other factors which may influence telomere length, e.g., body mass index, childhood maltreatment, socioeconomic status, physical activity [14], and current life stress [37]. In addition, although screening of psychiatric diseases was performed by self-report and by interviews, the possibility that sub-threshold disorders such as cigarettes and alcohol uses and unhealthy eating might affect telomere length in the present sample cannot be excluded entirely. Therefore, in further studies on the relationship between telomere length and interpersonal sensitivity, the assessment of these factors is needed.

## Conclusion

The present study suggests that subjects with higher interpersonal sensitivity have shorter leukocyte telomere length, implying that interpersonal sensitivity has an impact on cellular aging.

## Abbreviations

HPA: Hypothalamic-pituitary-adrenal
IPSM: Interpersonal Sensitivity Measure
